# Effect of real-world fear on risky decision-making in medical school-based students: A quasi-experimental study

**DOI:** 10.3389/fnbeh.2023.1030098

**Published:** 2023-03-02

**Authors:** Lei Wang, Sheng Chen, Wei Xiao

**Affiliations:** ^1^Department of Medical Psychology, Strategic Support Force Medical Center, Beijing, China; ^2^Department of Military Medical Psychology, Air Force Medical University, Xian, China

**Keywords:** risky decision-making, fear, calm, cortisol, balloon analog risk task, Cambridge gambling task, quasi-experimental study

## Abstract

**Objective:** To explore the effect of real-world fear on risky decision-making under certainty and uncertainty.

**Methods:** This quasi-experimental study enrolled non-psychology undergraduate volunteers aged between 17 and 20 years old from the Preventive Medical Institute medical school in Xi’an. Participants were randomly divided into two groups, and each group received a two-stage crossover design intervention (of a calm and fearful situation) and completed the tasks of risky decision-making under uncertainty (the balloon analog risk task: BART) and certainty (the Cambridge gambling task: CGT), respectively. The primary outcomes included the behavioral impulsivity measured by the BART value, and the speed of decision-making, the quality of decisions, the adventure index, behavioral impulsivity, and risk adjustment measured by CGT. The secondary outcome was the concentration of cortisol in the saliva.

**Results:** A total of 60 questionnaires and data were obtained from 60 participants (28 males and 32 females, aged 19.55 ± 0.75). Compared with the calm situation, participants were more likely to have a lower BART value (*p* = 0.013), slower speed of decision-making (*p* < 0.05), and higher adventure index (*p* = 0.018) in the fearful situation. The quality of decisions (*p* = 0.189), behavioral impulsivity index (*p* = 0.182), and risk adjustment (*p* = 0.063) between subjects in the fearful and calm situations were comparable. Furthermore, the mean value of the adventure index of CGT in male subjects was significantly higher than that in female subjects (*p* < 0.05), and the cortisol concentration in saliva during the fearful situation was significantly higher compared to the calm situation (*p* < 0.05).

**Conclusion:** Fear might reduce behavioral impulsivity under uncertainty, and increase the adventure index under certainty in risky decision-making. Risky behavior might be influenced by gender: under certainty in risky decision-making, men were more adventurous. Additionally, fear increased the secretion of cortisol in saliva.

## Introduction

Risky decision-making is a common research topic that effectively combines elements of psychology, behavioral science, biology, and economics (Andrews et al., 2018; Markkula et al., 2021). In decision theory, decision-making can be divided into being under certainty or uncertainty. Humans have the ability to predict probability under various conditions in tasks with unequal probability (Norton et al., 2019). Furthermore, decision-making under uncertainty is ubiquitous in daily life, where decisions can be made in probabilistically fuzzy situations. It is widely accepted that the response patterns of the human mind, adaptation to different environments, and certain mental and neurological diseases can influence risky decision-making abilities (Norbury et al., 2013; Goschke, 2014; Burns et al., 2020). People’s decision-making under risk generally share common tendencies, but there also exist substantial individual differences.

Emotions are capable of influencing human thoughts and actions. Decision-making is often influenced and biased by emotions, such as happiness, sadness, fear, and anger (Phelps et al., 2014; Hsieh, 2020). Moreover, affective experience is dynamic and continuously influenced by the encountered information in a decision-making context (Asutay and Västfjäll, 2022). Emotions are composed of multi-dimensional cognitive appraisals. The dominant evaluation latitude is called the core appraisal theme of emotions, and it has different dimensions of high and low certainty. Under inconsistent conditions, the appraisal tendency stimulated by emotions may be regulated by the attributes of risk. Therefore, the relationship between affect and a decision is complex. Notably, human decision-making often occurs under stressful conditions (Porcelli et al., 2012). It has been reported that fear serves as a basic experienced emotion, contributing to the avoidance of threats and also encouraging risk-taking (Chierchia et al., 2021). Previous studies have also demonstrated that fear was associated with both a subsequent increase and decrease in risk-taking (Lerner and Keltner, 2001). Nevertheless, the relationship between risky decision-making under certainty or uncertainty and fear remains unclear. More research is urgently needed to further reveal the underlying mechanisms of the impact of fear on risky decision-making.

Herein, a high-altitude glass platform at a TV tower was used to induce a fearful situation and landscape video clips in a laboratory were used to induce a calm situation. The combination method of an emotion self-report based on a psychological index and the salivary cortisol change based on a physiological index was utilized. This study aimed to investigate the difference in the effect of fear on risky decision-making under certainty and uncertainty.

## Method

### Study design and subjects

This quasi-experimental study enrolled 60 non-psychology undergraduates from the Preventive Medical Institute medical school in Xi’an aged between 18 and 20 years old who volunteered to participate.

All subjects were healthy and right-handed with normal or corrected-to-normal vision and had no color blindness. Moreover, none of them had a history of taking glucocorticoids, mental health diseases, or nervous system damage, nor had they participated in similar or related studies. For three days prior to the study, all subjects were tasked to ensure normal rest and sleep, and did not drink anything with caffeine or take central nervous system drugs. Written informed consent was obtained from all subjects before the study, and redeem the corresponding gifts according to the task achievement level. The study protocol was reviewed and approved by the ethics committee of the Air Force Medical University, Xi’an.

### Intervention

The different stages refer to the created situations. Stage A represented the calm situation. During this stage, the subjects in our department arrived in the laboratory and rested for half an hour. Subsequently, 8–9 min of natural scenery video footage without emotional content was reviewed to induce a calm mood, as has been recognized in the field. Stage B represented the fearful situation, which was designed to induce dread. Subjects were asked to remain on a 160-meter-high glass platform at a TV tower for half an hour to induce dread and maintain the persistence and ecological effects of the emotion based on the assumption that humans are hard-wired to fear heights (Menzies and Clarke, 1993). Lastly, the subjects filled out an emotional self-assessment scale.

The subjects were randomly divided into two groups with an allocation ratio of 1:1. One group experienced stage A first and then stage B, and the other group experienced stage B first and then stage A. There was a three-week washout period between two stages. The first samples of saliva were collected after the subjects entered the TV tower lounge for half an hour, after which subjects took the elevator to the glass platform 160 meters above the ground. Five minutes later, subjects filled out the self-rating mood scale, and the second saliva samples were collected. Then, the two tasks were performed and the third saliva sample was collected 15–20 min after the two tasks.

#### Task 1

The balloon analog risk task (BART), also known as ambiguous decision-making, is a computerized decision-making test to assess risk-taking under uncertainty. Indeed, BART has been applied to a number of studies on decision-making internationally. The dependent variable is the total number of inflating balloons/number of unexploded balloons (BART value). The operation interface is shown in Supplementary Figure 1. BART was written in computer programming (C++) and the simulation materials were presented on a computer screen *via* computer software. All subjects inflated 30 simulated balloons with preset random explosion points in advance, and accrued ¥2 as reward for each successive “pump” to make the balloons bigger. The bigger the balloons were blown, the more money rewarded. This reward system encouraged subjects to pay attention to the trade-off between rewards rather than simply risky inflating (Lerner and Keltner, 2001). The behavioral impulsivity of the subjects was measured by the BART value.

#### Task 2

The Cambridge gambling task (CGT) is a decision-making test under certainty to evaluate risk-taking behavior. CGT is mainly used to observe how people make decisions based on predictable rewards and punishments. The dependent variables include mainly the adventure index, impulse index, and speed of decision making. The operation interface of CGT is shown in Supplementary Figure 2. During this task, subjects could find 10 small boxes with two colors (red and blue) on the screen of the computer, one of which contained a special square with a yellow token. The subjects were asked to guess which color of boxes contained the yellow token, and then choose the color of the boxes. There were nine ratios of red and blue boxes, including 9:1, 8:2, 7:3, 6:4, 5:5, 4:6, 3:7, 2:8, and 1:9, and five kinds of stakes in the proportion to the subject’s principal, including 5%, 25%, 50%, 75%, and 95%. The bets were randomly displayed on the screen in ascending or descending order with a total of 36 bets. Each subject had 100 pieces of gold at the start of task. Since the ratio of the number of red or blue boxes for each bet changed randomly, each decision-making had different risks, the subjects needed to adjust the mean value of each bet to maximize the number of gold pieces. In order to avoid the impact of the exercise on cortisol secretion, any slightly strenuous activities were prohibited after lunch.

### Emotional self-rating

The emotional self-rating scale reported by Druckman and McDermott was used, containing 19 independent variables, such as: nervous, no feeling, scared, angry, fearful, happy, depressed, pessimistic, mad, disgusted, and nauseated (Asutay and Västfjäll, 2022). Subject self-report was adopted. According to Lerner’s practice, the emotions were classified as dread (fear, tension, scare), neutral, happy, sad, disgust, and anger (Porcelli et al., 2012). The degree of each emotion was determined according to the Likert 7-point scale (1 meant “extremely slight” and 7 meant “very strong”). Considering that the incidental emotions manipulated by the experiment had an impact on subsequent decision-making and were not easy to subside in a short period of time (Chierchia et al., 2021), only one emotional self-assessment was set before the two tasks in this study.

### Assessment of cortisol concentration in saliva

The higher the level of cortisol in the saliva, the higher the body’s sensitivity to stress. In this state, cortisol generally peaks after 10–30 mins. We collected the samples at 20–25 min after stimulation. According to the circadian rhythm of cortisol, the samples were selected around 3–4 pm. Before collecting saliva, subjects were asked to confirm fasting and maintain oral hygiene. Approximately 2–3 ml of mixed saliva was collected with a chewing swab. The supernatant was stored in a 20°C refrigerator, followed by centrifugation at 3,000 rotations/min for 10 min. Competitive inhibition enzyme-linked immunosorbent assay was used to determine the concentration of cortisol in saliva with an Epoch full-wavelength automatic enzyme label instrument (BioTek enzyme label instrument company, USA). Briefly, this included performing one instance of coating and sample addition, three instances of incubation, three of washing, the adding of antibodies, enzyme conjugates, and chromogenic substrates, and terminating the reaction to determine the result (specific steps are shown in the Supplementary Material). The main experimental consumables included: 1,000 μl pipettes (KE0037273), 200 μl pipettes (YE3K030591), 50 μl pipettes (DS35110), and 10 μl pipettes (KE0012951).

### Outcomes

The primary outcomes included the behavioral impulsivity measured by BART and the speed of decision making, quality of decisions, adventure index, behavioral impulsivity, and risk adjustment measured by CGT. The secondary outcome was the concentration of cortisol in saliva. The behavioral impulsivity measured by BART was calculated by the total number of inflating balloons/number of unexploded balloons (BART value). The speed of decision-making referred to the mean time of choosing the gold hidden in the box. The quality of decisions was measured by the number of choices of color selected the most times (the ratio of five red box colors to five blue box colors was not included in the analysis). The adventure index was the mean value of each bet (%). The behavioral impulsivity measured by CGT was the mean value of the bets in the descending order/the mean value of the bets in the ascending order (%). Risk adjustment referred to the ratio of bets invested by the subjects to the current gold owned.

### Statistical analysis

SPSS 20.0 (IBM Corp., Armonk, N.Y., USA) software was used for all data analysis. The comparison of the six emotional variables was analyzed by the Kruskal-Wallis test and the Nemenyi test. The impulsivity index and adventure index of the CGT were analyzed by paired sample Wilcoxon test. The concentrations of cortisol in saliva in the two groups were repeatedly measured and analyzed by repeated measures analysis. *Post-hoc* tests were used to compare the concentrations of cortisol in saliva between the two groups at different time points. Two-factor ANOVA was used to analyze the difference in the adventure index of male and female subjects in fearful and calm states, and the BART scores of male and female subjects in fearful and calm situations. Paired sample t test was used for comparison of the speed of decision making, quality of decisions, and adventure index in fearful and calm states during the CGT task. P values less than 0.05 were considered to be statistically significant.

## Results

**Figure 1:**
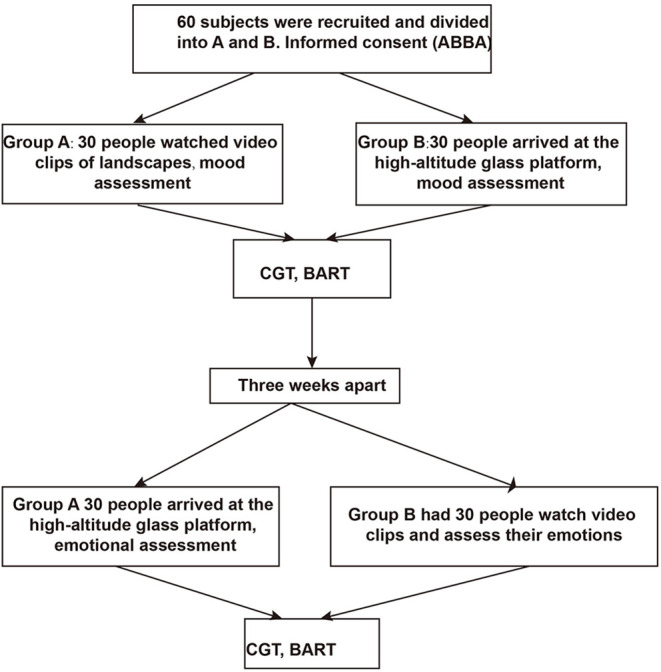
Experimental design process.

A total of 60 entire questionnaires and data were obtained from 60 subjects (28 male, 32 females, mean age of 19.55 ± 0.75), all of them university students (Table 1), and 4 invalid pieces of data were excluded due to missing data and effects of the female menstrual cycle. The experimental design process is shown in Figure 1.

**Table 1:** Basic characteristics.

**Variables**	***n* (%)**
Age	18–20 (100)
Sex	
Females	32 (53.3)
Males	28 (46.7)
Education	
University	60 (100)

**Figure 2:**
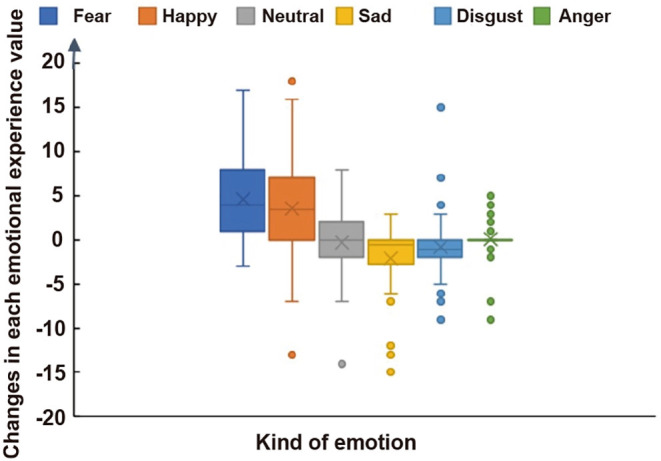
Comparison of six emotional experiences.

The fearful situation versus the calm situation, the change value of dread higher than in the calm situation 4.583 ± 4.208, and the change value of happiness increased by 2.767 ± 4.698, while the values of neutral and sad decreased. There was a significant difference between fear and each emotion (*p* < 0.001; Figure 2, Table 2). Moreover, there was significant difference in dread emotions between the subjects in the fearful situation and the calm situation (*p* < 0.05, Cohen’s *d* = 1.312), and the happy (excited) emotion in the fearful situation was significantly increased.

**Table 2:** Changes of six emotions (*n* = 60).

**Types of emotion**	**d**		**P**
	**mean ± SD**	**IQR**	
Dread	4.583 ± 4.208	4 (7)	0.001**
Happy	2.767 ± 4.698	2.5 (7)	
Neutral	−0.283 ± 4.162	0 (5)	
Sad	−2.017 ± 3.610	0 (2)	
Disgust	−0.733 ± 3.230	−1 (2)	
Anger	0.050 ± 1.899	0 (0)	

Note: **p < 0.01. d is the difference between various emotional variables in the two situations (taking the calm situation as the reference value).

The BART value of the subjects in the fearful situation was significantly lower than that in the calm situation (11.372 ± 3.728 vs. 12.996 ± 3.801, *p* = 0.013; Table 3). Additionally, two-way ANOVA showed that the main effect of emotion was a significant difference between the fearful situation and the calm situation (*p* = 0.009), while the BART value under uncertainty between males and females were comparable (*p* = 0.117). The interaction between gender and emotion was also not a significant difference (*p* = 0.127; Table 4).

**Table 3:** Comparison of BART values between the fearful situation and calm situation (*n* = 60).

**Situation**	***n***	**Mean ± SD**	***p***	***Cohen’s d***
Calm	60	12.996 ± 3.801	0.013*	0.431
Fear	60	11.372 ± 3.728		

Note: *p < 0.05.

**Table 4:** Two-way ANOVA of BART scores under different emotions and gender variables.

**Source of difference**	**Degrees of freedom**	**Mean square**	***p***	***ES***
Emotion	1	85.193	0.009**	0.111
Gender	1	40.308	0.117	
Emotion*Gender	1	28.146	0.127	
Error	58	11.771		

Note: 1.**p < 0.01; 2. ES (partial Eta squared).

The speed of decision-making of the subjects in the fearful situation (2.812 ± 1.336) was significantly slower than that in the calm situation (2.456 ± 0.990; *p* < 0.05).

Compared with the calm situation, the adventure index of the subjects in the fearful situation was significantly increased (0.501 ± 0.162 vs. 0.437 ± 0.180, *p* = 0.018). There was no significant difference in the quality of decisions (*p* = 0.189), behavioral impulsivity index (*p* = 0.182), and risk adjustment (*p* = 0.063) of subjects between the fearful situation and the calm situation (Table 5).

**Table 5:** Intra-group comparison of the CGT in the fearful situation and calm situations [*n* = 60, mean ± SD/ Md (QR)].

**Index of task**	**Fear (High Altitude Platform)**	**Calm (Situation video)**	***P***	***Cohen’ s d***
Speed of decision-making, min	2.812 ± 1.336	2.456 ± 0.990	0.048*	0.302
Quality of decisions	28.510 ± 4.970	27.680 ± 4.998	0.189	-
Adventure index	0.501 ± 0.162	0.437 ± 0.180	0.018*	0.376
Impulse index	0.151 (0.297)	0.154 (0.417)	0.057	-
Risk-adjustment.	1.686 (7.529)	2.169 (5.725)	0.182	-

Note: *p < 0.05.

Furthermore, the mean value of the adventure index of CGT in male subjects was significantly higher than that in female subjects (0.512 ± 0.170 vs. 0.432 ± 0.169, *p* < 0.05; Table 6). Additionally, two-way ANOVA showed that the main effects of emotion were significantly different in the adventure index of CGT between the calm situation and the fear situation (*p* = 0.021) as well as gender (*p* = 0.025). The interaction between gender and emotions was not significant (*p* = 0.408; Table 7).

**Table 6:** Analysis of the effect of gender on the adventure index of the Cambridge game task.

**Gender**	***n***	**Adventure index, mean ± SD**	***p***
Male	55	0.512 ± 0.170	0.12*
Female	64	0.432 ± 0.169	

Note: *p < 0.05.

**Table 7:** Two-way ANOVA of CGT under different emotion and gender variables.

**Source of difference**	**Degrees of freedom**	**Mean square**	***p***	***ES***
Emotion	1	0.115	0.021	0.088
Gender	1	0.189	0.025	0.084
Emotion*Gender	1	0.014	0.408	
Error	58	0.028		

Note: 1. *p < 0.05; 2. ES (partial Eta squared).

The ANOVA showed a significant difference in cortisol concentration among the three different collection times (*p* = 0.001). *Post-hoc* tests showed that the cortisol concentration in saliva from the second and third collections were comparable (*p* > 0.05), whereas the cortisol concentration in saliva from both the second and third collections were significantly higher when compared to the first collection (*p* < 0.05; Table 8).

**Table 8:** Concentrations of cortisol in saliva collected three times before and after manipulation of the fearful situation (nmol/L, mean ± SD).

**Time point of collection**	**Mean ± SD**	***p***
First collection	3.769 ± 3.506	0.001**
Second collection	5.562 ± 4.157	
Third collection	5.240 ± 3.869	

Note: **p < 0.01 refers to the Greenhouse-Geisser correction coefficient in the univariate analysis (also refers to the multivariate analysis). The first collection was half an hour before boarding the platform. The second collection was when the platform was boarded for 5 min, and the third collection was the end of the task.

## Discussion

The results suggested that fear might reduce the behavioral impulsivity under uncertainty, and increase the adventure index under certainty in risky decision-making. Risky behavior might be influenced by gender: under certainty in risky decision-making, men were more adventurous than women. Additionally, fear increased the secretion of cortisol in saliva.

Fear is an essential coping strategy under stress (Harb and Taylor, 2015). The subjects were placed on a high-altitude glass platform to immerse themselves in a fearful situation, which successfully induced fear and overcame any shortcomings of short timeliness of emotional induction. Cortisol is a stress hormone and released through the circulatory system into the saliva when the body is in a state of stress flow (Siart et al., 2017). In this study, the concentration of cortisol in the saliva of the subjects reached its highest peak just after reaching the high-altitude glass platform and had a downward trend as they adapted to the situation. The unpredictable and persistent stress stimuli led to a rise in cortisol upon reaching the high-altitude glass platform, indicating that the fear of heights achieved the desired effect.

A previous study has suggested that punishment sensitivity could influence human decision-making as well as emotion (Jean-Richard-Dit-Bressel et al., 2019). The excitement felt during the financial task could partially mediate the impact of fear on risk-taking, and thereby enhance individuals’ risk-taking behavior (Lee and Andrade, 2015). At the level of neural mechanisms, the brain regions related to feedback learning, punishment sensitivity, and the executive functioning of performing risky decision-making tasks include the dorsolateral prefrontal lobe, insula, anterior cingulate gyrus, orbitofrontal cortex, and parietal striatum (Helfinstein et al., 2014; Wei et al., 2016; Kohno et al., 2017). The increase of dopaminergic energy in the prefrontal cortex and striatum of the brain region under acute stress can make the acquisition of positive reward for probabilistic risky decision-making stronger than the learning of punishment (Mather and Lighthall, 2012; Lighthall et al., 2013). CGT has regularly been used to examine risky decision-making under ambiguity with relatively certain probability for potential rewards (Canale et al., 2017). Individuals took more risks with bets regarding probabilistic deterministic risky decisions and focused on potential higher short-term rewards when the odds were known (Brand et al., 2005). In this study, fear increased the adventure index of subjects compared to calming emotions, accompanied by a reduction in the average decision time in the CGT. In addition, male subjects were more likely to be inclined to risk-seeking than female subjects in both fearful and calm emotional situations, which was consistent with a previous study (Lei et al., 2021).

Sensory decision-making has been reported to involve making decisions under uncertainty (Norton et al., 2019). The probabilistic uncertainty of a task increases the uncertainty of the risk perception in the fearful situation, and promotes people to choose risk aversion. Under acute stress (including fear), the functional connections between the amygdala and the core brain regions of the prominence network (dorsal anterior cingulate gyrus and anterior insula) and the midbrain are significantly enhanced, which causes a hypervigilance state in subjects (van Marle et al., 2010). Therefore, it can be speculated that fear stress, as a low certainty, leads to a high-vigilance state, which prompts people to reduce impulsivity in the face of probabilistically uncertain risky decisions and thus choose risk avoidance. BART is widely used to assess risky decision-making tendencies with probabilistic uncertainty, which can predict real risk-taking behavior (Lejuez et al., 2007). It has been reported that total BART points were negatively associated with risky decisions (Addicott et al., 2015). Heilman et al. suggested that naturally occurring negative emotions influence risk-aversion in a task of decision-making under uncertainty (Heilman et al., 2010). Moreover, in this study, the BART value in the fearful situation was significantly lower than that in the calm situation. Accordingly, it can be speculated that the apparent fear affected the risk perception of subjects’ decision-making, which prompted the subjects to choose risk avoidance in the fearful situation. However, there was no difference in the effect of gender on risky decision-making tendency with uncertain probability, which was inconsistent with the results of the CGT.

Emotions ultimately affect decision-making through the deterministic dimension (Winterich et al., 2010; Lerner et al., 2015). The same emotion may have different effects on different decision-making tasks. Subjects experiencing fear opted for risk aversion when the certainty of risky decision-making tasks was low (uncertain probability). However, the effect of emotions on risk appetite was inhibited when the certainty of risky decision-making tasks was fixed (certain probability). Our results also suggested that emotions may interact with the attributes of the task. The whole experiment was designed in accordance with the ABBA crossover, which balances the practice effect, fatigue effect, and especially the sequence effect. The quasi-experiment effectively made up for the situation that the real experiment could not achieve, and is applicable in a wider range of situations.

However, this study had some limitations. The number of participants was limited. Moreover, the emotion-induction method presented in this study may be affected by related emotions, such as expected emotions, and self-control and impulsiveness. The mutual interference between two or more emotions needs to be effectively controlled in future research. Furthermore, additional “risky decision-making” tasks are needed to validate the conclusions drawn in this study.

In conclusion, fear might reduce behavioral impulsivity under uncertainty, and increase the adventure index under certainty in risky decision-making which might be influenced by gender, and, additionally, increases the secretion of the cortisol in saliva.

## Data availability statement

The original contributions presented in the study are included in the article/Supplementary material, further inquiries can be directed to the corresponding author.

## Ethics statement

The studies involving human participants were reviewed and approved and the study protocol was reviewed and approved by the ethics committee of the Air Force Medical University. Written informed consent to participate in this study was provided by the participants’ legal guardian/next of kin.

## Author contributions

LW and WX contributed to the study conception and design. All authors collected the data and performed the data analysis. All authors contributed to the interpretation of the data and the completion of figures and tables. All authors contributed to the drafting of the article and final approval of the submitted version.
