# Supplementary figures and images for: Effect of real-world fear on risky decision-making in medical school-based students: A quasi-experimental study
Source: Front Behav Neurosci. 2023 Mar 2;17:1030098. doi: 10.3389/fnbeh.2023.1030098 (PMC10017853; doi:10.3389/fnbeh.2023.1030098)

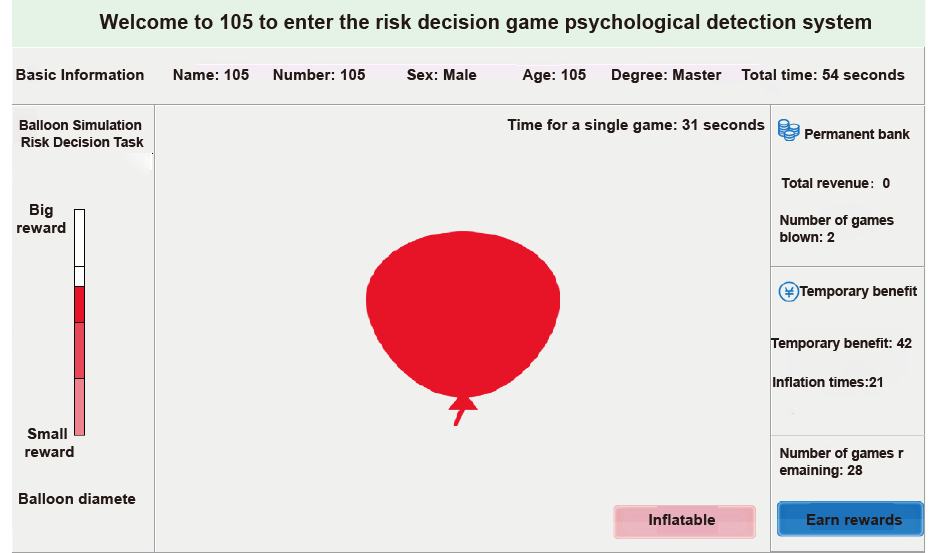

Supplement: Supplementary file 1 [file Image_1.tif]

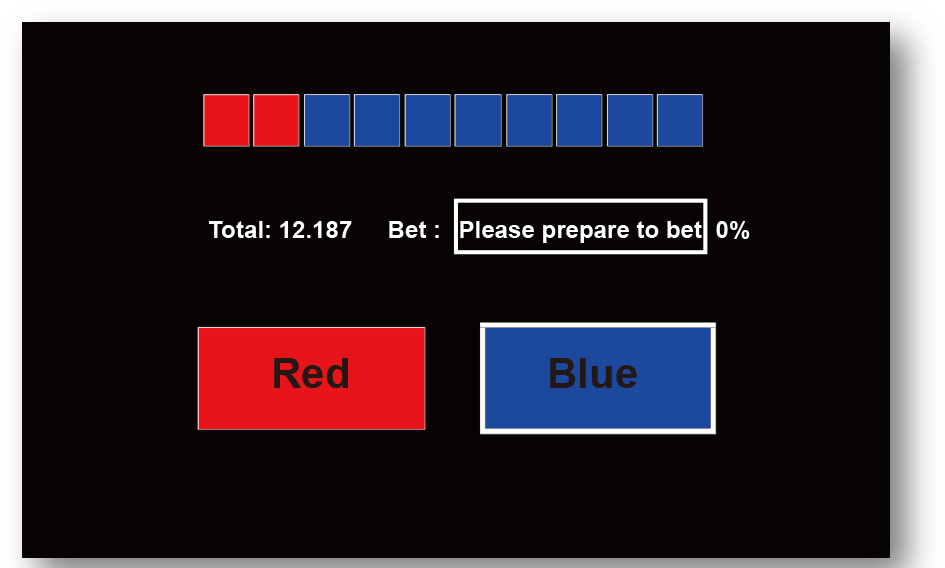

Supplement: Supplementary file 2 [file Image_2.tif]
